# Anaesthetists' current practice and perceptions of aerosol‐generating procedures: a mixed‐methods study

**DOI:** 10.1111/anae.15803

**Published:** 2022-07-21

**Authors:** A. J. Shrimpton, C. E. D. Osborne, J. M. Brown, T. M. Cook, C. Penfold, L. Rooshenas, A. E. Pickering

**Affiliations:** ^1^ Anaesthesia, Pain and Critical Care Sciences, School of Physiology, Pharmacology and Neuroscience University of Bristol UK; ^2^ Anaesthesia, Pain and Critical Care Sciences, School of Physiology, Pharmacology and Neuroscience University of Bristol UK; ^3^ Department of Anaesthesia and Intensive Care Medicine North Bristol NHS Trust Bristol UK; ^4^ Department of Anaesthesia and Intensive Care Medicine Royal United Hospital NHS Trust Bath UK; ^5^ NIHR Bristol Biomedical Research Centre University Hospitals Bristol and Weston NHS Foundation Trust and University of Bristol UK; ^6^ Bristol Medical School, Bristol Population Health Science Institute University of Bristol UK; ^7^ Anaesthesia, Pain and Critical Care Sciences, School of Physiology, Pharmacology and Neuroscience University of Bristol UK

**Keywords:** aerosol generating procedures, anaesthetists' perception, national infection control and prevention guidelines, respiratory protective equipment

## Abstract

The evidence base surrounding the transmission risk of ‘aerosol‐generating procedures’ has evolved primarily through quantification of aerosol concentrations during clinical practice. Consequently, infection prevention and control guidelines are undergoing continual reassessment. This mixed‐methods study aimed to explore the perceptions of practicing anaesthetists regarding aerosol‐generating procedures. An online survey was distributed to the Membership Engagement Group of the Royal College of Anaesthetists during November 2021. The survey included five clinical scenarios to identify the personal approach of respondents to precautions, their hospital's policies and the associated impact on healthcare provision. A purposive sample was selected for interviews to explore the reasoning behind their perceptions and behaviours in greater depth. A total of 333 survey responses were analysed quantitatively. Transcripts from 18 interviews were coded and analysed thematically. The sample was broadly representative of the UK anaesthetic workforce. Most respondents and their hospitals were aware of, supported and adhered to UK guidance. However, there were examples of substantial divergence from these guidelines at both individual and hospital level. For example, 40 (12%) requested respiratory protective equipment and 63 (20%) worked in hospitals that required it to be worn whilst performing tracheal intubation in SARS‐CoV‐2 negative patients. Additionally, 173 (52%) wore respiratory protective equipment whilst inserting supraglottic airway devices. Regarding the use of respiratory protective equipment and fallow times in the operating theatre: 305 (92%) perceived reduced efficiency; 376 (83%) perceived a negative impact on teamworking; 201 (64%) were worried about environmental impact; and 255 (77%) reported significant problems with communication. However, 269 (63%) felt the negative impacts of respiratory protection equipment were appropriately balanced against the risks of SARS‐CoV‐2 transmission. Attitudes were polarised about the prospect of moving away from using respiratory protective equipment. Participants' perceived risk from COVID‐19 correlated with concern regarding stepdown (Spearman's test, R = 0.36, p < 0.001). Attitudes towards aerosol‐generating procedures and the need for respiratory protective equipment are evolving and this information can be used to inform strategies to facilitate successful adoption of revised guidelines.

## 1 Introduction

When SARS‐CoV‐2 emerged in 2019, it spread rapidly and overwhelmed healthcare services in Wuhan and parts of northern Italy. The predominant modes of disease transmission were thought to be via droplets >5 μm diameter and fomites. Airborne transmission was only considered to occur during medical interventions classified as aerosol‐generating procedures (AGP) [1]. Medical practitioners with advanced airway skills were deemed to be at very high risk of contracting COVID‐19 due to their performance of AGPs [2]. This message was forcefully imparted on the anaesthetic workforce and the importance of wearing airborne protection personal protective equipment (PPE) for all AGPs was reinforced [3].

Our understanding of the viral dynamics and patterns of SARS‐CoV‐2 transmission has increased substantially over the course of the pandemic, including the recognition that airborne transmission occurs in the absence of AGPs [4, 5]. Emerging clinical aerosol evidence has demonstrated that many AGPs generate less aerosol than natural respiratory activities, such as breathing and coughing [6, 7, 8, 9, 10, 11], and epidemiological evidence indicates anaesthetists and intensivists may have a lower risk of infection and hospitalisation compared with other frontline healthcare workers [12]. The AGP framework likely impacts on healthcare efficiency and quality, and presents a challenge to addressing the backlog of patients waiting for elective surgery, which in the UK exceeds 6 million people [13]. With the accumulation of evidence around aerosol generation risk from AGPs, there is likely to be a reappraisal of UK infection prevention and control (IPC) guidance.

Changes to guidance may be welcomed by some healthcare workers but generate anxiety in others and this may lead to issues with rollout and implementation. The views of practicing anaesthetists towards existing AGP guidance and possible revision are unknown. This study aims to explore perceptions about the management of AGPs, attitudes to potential guideline alterations and consequent practice change.

## 2 Methods

This was a mixed‐methods study, comprising a survey disseminated to UK anaesthetists followed by qualitative interviews with purposefully selected survey respondents to explore their perceptions in greater depth. The Health Services Research Committee Executive Management Board of the Royal College of Anaesthetists reviewed the survey before circulation. Ethical approval for the qualitative component was granted by the University of Bristol Faculties of Life Sciences and Science Research Ethics Committee.

The online survey, implemented using KoBoToolbox (https://www.kobotoolbox.org), was developed iteratively through two pilots. The initial draft was distributed to the members of the University of Bristol Anaesthetic Pain and Critical Care research group. A second iteration was distributed to the members of the Severn Trainee Anaesthetic Research Group. In each case, responses and feedback helped inform the final survey design. The final survey was distributed by the Royal College of Anaesthetists via email to their Membership Engagement Panel, a cohort of members at all career stages whose views contribute to the direction and strategy of the College via regular updates and surveys [14, 15]. The survey was open for responses between 17 November 2021 and 5 December 2021 and a reminder email was sent 4 days before survey closure (online Supporting Information, Appendix S1).

The anonymous survey included five clinical scenarios that sought to identify respondents' hospital policies, their personal views on precautions required during AGPs and the impact of precautions on healthcare delivery (Box 1). The scenarios were designed to explore the management of patients with confirmed positive, indeterminate or confirmed negative SARS‐CoV‐2 infection during interactions classified as AGPs and non‐AGPs at the time of the survey. We refer to patients of indeterminate SARS‐COV‐2 status as ‘amber’. Under the prevailing UK guidance, only Scenario 3 (Box 1) is recognised as being an AGP. Droplet PPE was defined as follows: fluid‐resistant surgical mask (non‐respirator), apron, gloves and eye protection. Respiratory protective equipment (RPE) was defined as droplet PPE plus a fitted respirator type mask (FFP3/hood) and fluid repellent gown. Personal characteristics and perceived personal risk were sought to stratify responses and identify patterns.

Box 1 **Survey clinical scenarios.**

**Scenario 1:** Pre‐operative assessment of a patient confirmed to be infected with SARS‐CoV‐2 who is symptomatic.

**Scenario 2:** Pre‐operative assessment of an asymptomatic patient who has not self‐isolated and has a SARS‐CoV‐2 polymerase chain reaction test pending (amber).

**Scenario 3:** Tracheal intubation of the patient in scenario 2.

**Scenario 4**: Supraglottic airway device insertion for the patient in scenario 2.

**Scenario 5:** Tracheal intubation of a patient who had a negative SAR‐CoV‐2 polymerase chain reaction test, has self‐isolated for 14 days before admission and is asymptomatic for COVID‐19.

Responses were screened for duplicates before analysis. Descriptive analysis was performed using R for respondent characteristics, Likert scale responses and response rates (https://www.R‐project.org). Thematic analysis was performed on comments from the free‐text sections that allowed respondents to provide additional commentary [16]. Respondents were given the option to submit their email address if they consented to additional contact for qualitative interviews to discuss their perceptions in more detail. This qualitative component enabled exploration of perceptions in greater depth than could be done by the survey alone. Purposeful sampling was used to obtain an initial group of 12 participants that represented the spectrum of respondent characteristics and level of concern about AGPs. Further sampling was driven by the intention to ensure representation across the range of participant characteristics and to achieve data saturation. Following informed consent, semi‐structured interviews were conducted and recorded via web‐conferencing software by a single investigator (CO). A topic guide (designed by CO, AS, AEP and LR before interviewing) was piloted in an interview not included in the final dataset. The topic guide was used to ensure similar areas were explored across all interviews. Audio recordings were transcribed verbatim and imported into NVivo (V12; QSR International Pty Ltd., Daresbury, Cheshire) to support analysis.

Data were analysed thematically, using a grounded constant comparison method [17]. Data analysis occurred concurrent with data collection, to allow emerging findings to inform subsequent data collection efforts through iterations of the topic guide, which underwent four revisions. Individual features of the text (codes) were derived from the raw data inductively. Codes were iteratively developed into themes, by grouping together codes with similar or connected meaning. The process was iterative because theme names, sub‐themes and overarching themes evolved as analysis progressed and previously coded transcripts were re‐examined considering later data collection [16, 17, 18]. The study team met regularly throughout the process of data collection and analysis to discuss the development of the coding frame in line with emerging findings. Data saturation, defined here as the point at which no new insights arose from at least two consecutive interviews, was agreed by the team to have occurred after 18 interviews.

Several strategies were used to enhance the rigour of the qualitative work. Credibility was enhanced through analyst triangulation, whereby a sample of early transcripts was independently coded by two researchers (CO and AS) to check for consistency in interpretation and coding; minimal semantic discrepancy was identified. The entire process of analysis was overseen by an experienced qualitative methodologist (LR), who met with the qualitative researcher (CO) regularly to discuss refinements to data collection processes and interpretation of data. The main qualitative researcher (CO) also kept a reflective diary to document personal reflections and experiences to mitigate against these inadvertently shaping data collection and analysis. We also sought to identify ‘negative cases’ and report these explicitly to ensure we reported the different dimensions of accounts. Member‐checking was undertaken to ensure clarification and understanding of interviewee responses. This was performed by summarising what was said both during and at the end of the interviews. Reporting of the qualitative elements of this study was guided by the consolidated criteria for reporting qualitative studies checklist.

Survey results and qualitative findings are presented together, arranged according to the core topics investigated in the study; the emergent themes from the qualitative work are presented subsequently. The qualitative findings are supported by illustrative quotes (online Supporting Information, Tables S1–S4). Selected quotes are included in the text, interviewee identifiers are presented alongside quotes (Æ# represents an interviewed anaesthetist, FT represents free‐text comments from the survey).

## 3 Results

A total of 333 completed surveys were received and included in the analysis, representing an 8% response rate. Of these, 127 (38%) agreed to be contacted for the qualitative component of the study. In all, 18 interviews were undertaken between 31 December 2021 and 9 February 2022. The characteristics of all survey respondents and interviewees were compared with the 2020 RCoA Anaesthetic Workforce Census. This showed that our samples were broadly representative of the UK anaesthetic workforce across many domains (Table 1) [19]. The interview participants varied in terms of role (12 consultants; 3 Associate Specialists; 2 Registrars; 1 Core Trainee), their self‐reported perceptions of risk (as indicated in survey responses) and age (Table 1).

**Table 1:** Characteristics of survey respondents and the qualitative sample compared with characteristics from the medical workforce census undertaken by the Royal College of Anaesthetists [23]. RCoA census data consultants: n = 7537 for sex; n = 7959 for location. Anaesthetists in training: n = 3909. All workforce: n = 14,901.

	Subcategory	Survey respondents	Qualitative participants	Royal College of Anaesthetists Census
Consultants		196 (59%)	12 (67%)	7959 (53%)
Sex	Male	116 (60%)	6 (50%)	N/A (62%)
	Female	78 (40%)	6 (50%)	N/A (38%)
	Prefer not to say	2 (1%)	0	0
Age; y	30–39	23 (12%)	1 (8%)	1421 (19%)†
	40–49	82 (42%)	7 (59%)	3216 (43%)†
	50–59	65 (34%)	3 (25%)	2377 (32%)†
	60–69	23 (12%)	1 (8%)	506 (7%)†
	70+	0	0	17 (0%)†
	Prefer not to say	3 (1%)	0	0
Location	England	154 (79%)	8 (66%)	6471 (81%)
	Scotland	23 (12%)	2 (17%)	776 (10%)
	Wales	14 (7%)	2 (17%)	433 (5%)
	Northern Ireland	5 (3%)	0	279 (4%)
Trainees		93 (28%)	3 (17%)	3799 (26%)
Sex	Male	57 (63%)	2 (67%)	1921 (51%)
	Female	33 (37%)	1 (33%)	1878 (49%)
	Prefer not to say	3 (1%)	0	0
TOTAL		333	18	14901*
Sex	Male	195 (59%)	10 (56%)	−
	Female	131 (39%)	8 (44%)	−
	Prefer not to say	7 (2%)	0	−
Ethnicity	White	212 (64%)	12 (67%)	−
	Asian/Asian British	83 (25%)	4 (22%)	−
	Other ethnic group	16 (5%)	0	−
	Black/African/Caribbean/Black British	7 (2%)	2 (11%)	−
	Mixed/multiple ethnic groups	3 (1%)	0	−
	Prefer not to say	12 (4%)	0	−
Location	England	270 (81%)	13 (72%)	−
	Scotland	36 (11%)	3 (17%)	−
	Wales	18 (5%)	2 (11%)	−
	Northern Ireland	9 (3%)	0	−
Work	District General Hospital	158 (47%)	7 (39%)	−
	University Teaching Hospital	98 (29%)	7 (39%)	−
	Tertiary Referral Centre	75 (23%)	4 (22%)	−
	Private hospital/unit	1 (1%)	0	−
	Day case procedure centre	1 (1%)	0	−
Role	Consultant level doctor (or post CCT fellow)	196 (59%)	12 (67%)	8040 (54%)
	Registrar (ST3−ST8)	62 (19%)	2 (11%)	2562 (17%)
	Associate Specialist/SAS/LAS/LAT	27 (8%)	3 (17%)	1470 (10%)
	Core Trainee (CT1−CT3)	21 (6%)	1 (6%)	972 (7%)
	ACCS	10 (3%)	0	698 (5%)
	Clinical/Research Fellow/Trust Doctor	12 (4%)	0	840 (6%)
	MTI (Medical Training Initiative)	5 (2%)	0	146 (1%)
Age; y	20–29	23 (7%)	1 (6%)	−
	30–39	104 (31%)	2 (11%)	−
	40–49	103 (31%)	10 (56%)	−
	50–59	71 (21%)	3 (17%)	−
	60–69	25 (8%)	2 (11%)	−
	70–79	1 (0.5%)	0	−
	Prefer not to say	6 (2%)	0	−

N/A, not available; †out of 7537; *consultants = 8040.

**Figure 1:**
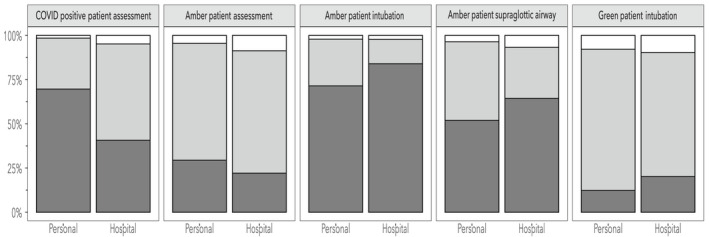
Hospital vs. personal choice of personal protective equipment for each scenario. Dark grey bar, respiratory protective equipment; light grey bar, droplet personal protective equipment; white, unsure. COVID positive, SARS‐CoV‐2 positive and symptomatic; Amber, asymptomatic patient of indeterminate SARS‐CoV‐2 status; Green, patient confirmed SARS‐CoV‐2 negative. n = 333 for personal and 312 for hospital.

Of the respondents, 329 (99%) had received two doses of a COVID‐19 vaccine as compared with 91% of all NHS healthcare staff for the same period [20]. Furthermore, 293 (89%) had also received a third vaccine dose. Regarding personal risk, 68 (20%) identified themselves as either clinically extremely vulnerable or at high risk from the effects of COVID‐19. Likewise, 62 (19%) contracted COVID‐19 before completing the survey. When undertaking a pre‐operative patient assessment, 220 (66%) reported wearing droplet PPE for a patient of indeterminate or ‘amber’ COVID‐19 status, which fell to 96 (29%) when the patient was confirmed as being SARS‐CoV‐2 positive (Fig. 1). Likewise, 69 (22%) reported that their hospital guidance was to wear RPE to undertake a pre‐operative assessment of an amber patient and 127 (41%) for a patient confirmed as positive for SARS‐CoV‐2.

Regarding tracheal intubation of an amber patient, 262 (84%) reported their hospital guidelines mandated the use of RPE, whereas it was the personal choice of 238 (71%) (Fig. 1). In contrast, 88 (26%) would use droplet precaution PPE, and 43 (14%) of their hospital guidelines mandated droplet precaution PPE only in this scenario. In those hospitals that required RPE for tracheal intubation, 154 (59%) reported ‘fallow time’ also being hospital policy. Of note, 63 (20%) worked in hospitals that mandated the use of RPE during tracheal intubation of a patient confirmed negative for SARS‐CoV‐2‐ and 41 (12%) also preferred RPE in this situation.

Similar proportions of respondents felt that pre‐operative assessment of a patient with confirmed SARS‐CoV‐2 infection and tracheal intubation of an amber patient require RPE, 232 (70%) and 238 (71%), respectively. Regarding the risk of SARS‐CoV‐2 transmission 190 (57%) judged this to be higher for tracheal intubation of an amber patient as compared with speaking with the same patient pre‐operatively. For insertion of a supraglottic airway device in an amber patient, 201 (64%) of respondents' hospital guidelines advised, and 173 (52%) of respondents preferred the use of RPE. Removal of these devices in the operating theatre was recommended in hospital policies for 167 (83%) of respondents and 106 (53%) reported the need for fallow times following supraglottic airway device removal.

### 3.1 Awareness and perceptions of guidelines

**Figure 2:**
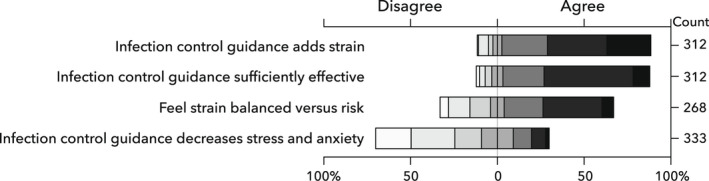
Degree to which infection prevention and control guidance affect work strain, stress and anxiety.

Of the respondents, 312 (94%) were aware of their hospital's IPC guidelines for AGPs and 264 (85%) felt this guidance prevented nosocomial SARS‐CoV‐2 transmission. Furthermore, 268 (86%) reported that guideline implementation added strain to working practices, yet 169 (63%) felt that this added strain was appropriately balanced against the risks of SARS‐CoV‐2 transmission (Fig. 2). Regarding RPE, 251 (75%) felt that it reduced their risk of contracting COVID‐19. However, 203 (61%) felt it did not decrease healthcare worker stress and anxiety. The interviews built on the survey findings by providing more insight into the perceptions of hospital IPC AGP guidelines and the reasons for adherence or non‐adherence. Most interviewees followed the AGP guidelines, even if their personal perception of the risk posed by anaesthetic procedures differed from the guidance*: “Am I rule follower or a rule breaker? I'm a rule follower, I think most of us aren't we at the bottom line”* (*Æ5)*.

In agreement with the survey, some interviewees felt protected by RPE; however, others complied as they felt their clinical leadership (Æ8) role necessitated following their hospital policies. Others followed the guidance to avoid conflict within the workplace or to appease the fears of concerned team members (online Supporting Information, Table S1, quotes 1–4). Those who did not follow the guidance felt their risk was low during the performance of AGPs and/or chose not to wear all components of RPE. Despite high rates of adherence to the IPC guidance as indicated in the survey, the interviews revealed concern about the extent to which guidance was evidence based. Interviewees expanded on this by giving examples of how guidance had not changed in alignment with evidence: *“I think [AGP guidelines are] very out of date for what the evidence is now around what is an AGP and what isn't an AGP” (Æ15)*.

Some interviewees expressed frustration at the lack of change to AGP guidelines since the early phase of the pandemic, despite the emergence of clinical aerosol evidence quantifying the relative risk of aerosol generation and a lower infection rate for anaesthetists compared with other frontline healthcare workers (such as those working on respiratory wards). Some also felt guidance did not account for important contextual issues, such as the introduction of rapid, sensitive screening techniques for SARS‐CoV‐2 and increasing rates of vaccination among staff and patients. These factors resulted in a loss of trust in the ‘IPC decision makers’ (online Supporting Information, Table S1, quotes 5–7). Some interviewees opined *“there's very much a guideline culture, particularly around COVID‐19”* (Æ10), which has arisen from the pandemic and is difficult to challenge. Others suggested the disconnect between the static IPC guidance and the evolving pandemic has led to the observed variation in practice at both hospital and individual levels that was noted in the survey (online Supporting Information, Table S1, quotes 7–9).

Interviewees had divergent perspectives on the concept of AGPs. Tracheal intubation was the most contentious as to whether it was high risk and whether it should be classed as an AGP. Some interviewees believe tracheal intubation carries a higher risk of SARS‐CoV‐2 transmission than speaking to a patient pre‐operatively. However, many felt this increased risk was not from the procedure but from being near the patient, and their airway. Others felt administering neuromuscular blocking drugs reduced the risk of aerosol generation as the patient was then unable to breathe or cough (online Supporting Information, Table S1, quotes 10–16). Most felt the infectious status of the patient was more relevant than the procedure itself: “*If you don't have COVID you're no risk to me at all” (*Æ13).

Coughing was universally agreed to be high risk for aerosol generation, and many perceived being near a coughing patient with SARS‐CoV‐2 infection as higher risk than performing tracheal intubation (online Supporting Information, Table S1, quotes 17–20). Along similar lines, several interviewees perceived tracheal extubation, use of nebulisers, bronchoscopy and endoscopy to be high risk for aerosol generation and felt these should remain classified as AGPs because they often induce coughing.

### 3.2 Impact of guidelines on practice

**Figure 3:**
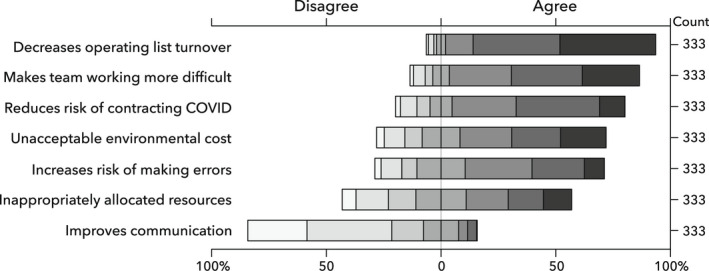
Impacts of using respiratory protection equipment and infection prevention and control precautions.

In the survey, 305 (92%) stated that the use of RPE and fallow times decreased operating list turnover, 376 (83%) felt RPE made team working more difficult and 255 (77%) felt it did not help communication. Furthermore, 202 (61%) stated RPE increased the risk of making clinical errors and 212 (64%) felt its use constituted an unacceptable environmental cost (Fig. 3). There was a divergence in respondents' views about whether current IPC guidance represents an appropriate allocation of resource, 153 (46%) vs. 107 (32%), respectively. Expanding on the survey findings, all interviewees discussed experiences of communication being impeded by RPE, which caused significant distress for some respondents*: “It was quite uncomfortable then with the loud sound, you are shouting at each other, and you know…so… it was like quite distressing at times”*. (Æ14).

The wearing of RPE was reported to negatively impact the development of patient rapport, communication with other team members and specifically to either increase the risk of, or directly contribute to, critical incidents (online Supporting Information, Table S2, quotes 1–5). The interviewed respondents confirmed the use of RPE had reduced the efficiency of anaesthetic care, some were frustrated by this impact and felt that the trade‐off between productivity and IPC guidance was not appropriately balanced*: “Our productivity has probably halved, probably worse than that. It's made massive changes to … our day‐to‐day working lives”* (Æ5).

A small number felt that the IPC guidance was totally disproportionate to the risk and would welcome a step down in RPE; they stated it did not convey extra protection as they felt their activities were not high risk for SARS‐CoV‐2 transmission. However, a few also reported a benefit of *“protected fallow time”* after tracheal intubation to ensure their anaesthetic preparation was optimised before draping and surgery commenced (online Supporting Information, Table S2, quotes 6–8). Most interviewees made a distinction between the use of FFP3 masks and the other components of RPE, such as gowns, visors and the use of multiple gloves. During AGPs, FFP3 masks were consistently worn but lower compliance was reported for other components of RPE. For example, most interviewees had stopped wearing visors as they felt their use impeded clinical practice to such an extent it posed a risk to both patient care and safety, with little discernible benefit to the wearer (online Supporting Information, Table S2, quotes 9,10). Despite reporting difficulties intrinsic to RPE use, many felt protected by the precautions, and some were happy to wear RPE for prolonged periods (online Supporting Information, Table S2, quotes 11,12). A frequent comment was that anaesthetists felt better protected than some of their other healthcare colleagues due to having better access to RPE (online Supporting Information, Table S2, quotes 13–16). Among those interviewees who did not identify as being at high risk from COVID‐19, there was recognition of the importance of RPE for those anaesthetists that do (online Supporting Information, Table S2, quote 17).

### 3.3 Personal perception of risk

*“I felt that as doctors we are trained to treat patients whatever may be their disease, so we just have to take the necessary precautions”* (FT152).

**Figure 4:**
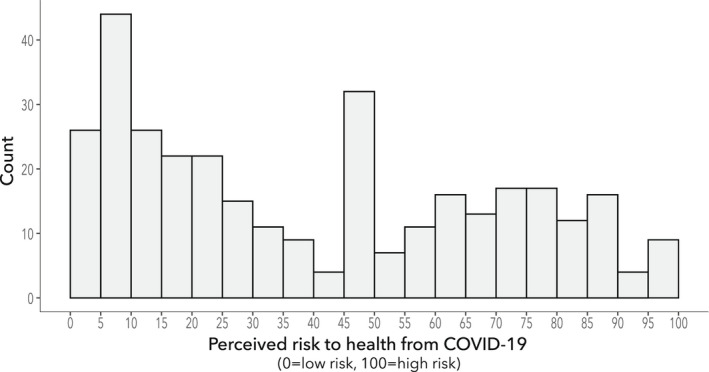
Perception of personal risk from COVID‐19. If vaccinated, this is the perceived risk before vaccination. n = 333.

**Figure 5:**
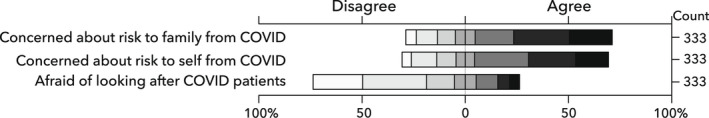
Concerns regarding risks from COVID‐19.

The survey showed there was a bimodal pattern of perceived personal risk from COVID‐19 with distinct groups centred around low risk and high risk (Fig. 4). Younger respondents tended to perceive the risk to their personal health from COVID‐19 to be lower than older anaesthetists, RR = 0.38, p < 0.001, 95%CI 0.28–0.47. Despite 221 (66%) being concerned about the risk from COVID‐19 to both their own and 228 (68%) to their family's health, there was a striking lack of fear regarding caring for patients with COVID‐19, with 228 (68%) not fearful (Fig. 5 and online Supporting Information, Table S3, quote 1). During the interviews, the main factors mentioned that influenced personal risk perceptions were: ethnicity; age; sex; family situation; personal medical history; and pandemic experience. Many felt the formal hospital risk assessments did not accurately reflect their individual risk. These respondents often conducted their own ad hoc risk assessments for donning RPE if they felt the risk posed to them was high, which accounted for some of the variance in practice reported in the survey (online Supporting Information, Table S3, quote 2–4)*: “I follow generally what the hospital wants except that if I particularly feel that I'm wearing full PPE, that's what I'm doing. And if anyone wants to argue with me, they can do it”. (*Æ*18)*.

Those anaesthetists with a higher perceived risk often had family members or friends who had experienced severe COVID‐19 symptoms (online Supporting Information, Table S3, quotes 5,6). While some clinicians felt reassured after they had experienced COVID‐19 and then recovered uneventfully from the illness, others were experiencing long COVID symptoms which influenced their views (online Supporting Information, Table S3, quotes 7,8). Some interviewees were unconcerned by the prospect of contracting SARS‐CoV‐2 and viewed it more as an inconvenience. However, a small number of anaesthetists were still concerned about serious morbidity or death demonstrating the spectrum of risk perception (online Supporting Information, Table S3, quotes 9,10). The focus of concern among most interviewees has evolved from dying from COVID‐19 to the consequences of living with symptoms of long COVID and the impact on other aspects of family life (online Supporting Information, Table S3, quotes 11,12). Many interviewees felt they were more likely to contract COVID‐19 outside the workplace from either their own children or during social interactions, which supports the survey findings that most anaesthetists felt relatively safe at work (online Supporting Information, Table S3, quotes 13–15).

### 3.4 Attitudes to change in guidance

**Figure 6:**
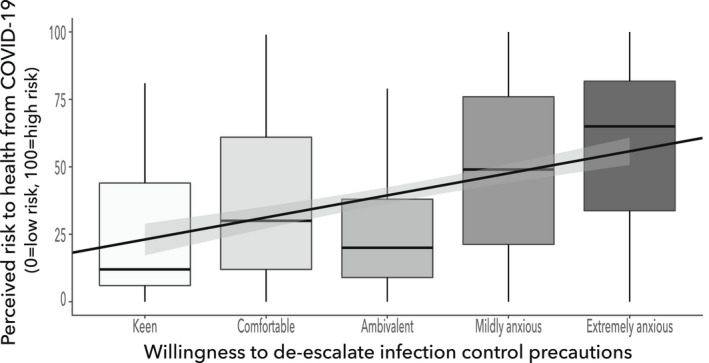
Willingness to de‐escalate respiratory protection equipment vs. perceived risk to self from COVID‐19. Regression line overlaid with 95%CI. Intercept = 14.9, slope = 8.2, R^2^ = 0.13, F(1, 331) = 49.65, p < 0.001.

Of those surveyed, 112 (34%) were comfortable or keen to move away from using RPE during airway management of an amber patient, but 168 (51%) remained anxious about the potential de‐escalation of IPC precautions, with 58 (17%) extremely anxious about this prospect. The degree of anxiety regarding de‐escalation had a positive correlation with perceived personal risk from COVID‐19; RR = 0.36, p < 0.001, 95%CI 0.26–0.45 (Fig. 6).

### 3.5 Theme 1: change in anaesthetic practice

*“A lot of people [adapted] their practices to try to circumvent AGPs… it's been very, very confusing for me”* (Æ*9*).

Many reported the pandemic and IPC guidelines had changed their practice. Some performed more ‘deep’ tracheal extubations and supraglottic airway device removals to reduce the risk of coughing, while others attempted to avoid AGPs entirely by using more regional or awake techniques. Supraglottic airway device use was more polarised with some avoiding their use as they felt the airway was not secure which was perceived to carry a higher risk of aerosol generation. However, others used supraglottic airway devices more frequently as their use has never been defined as an AGP. The increased use of videolaryngoscopy was frequently mentioned in both the free‐text comments and during interviews, and many interviewees stated they performed more rapid sequence inductions to avoid facemask ventilation (online Supporting Information, Table S4, quotes 1,2).

### 3.6 Theme 2: change in perceptions over time

*“The Italian intensivists were seen to be falling apart on live TV almost and that was shocking and greatly concerning and we also thought this terrible thing was coming to us and no one really knew what your personal risk was going to be” (*Æ*5)*.

Underpinning perceptions of risk identified by the survey, many interviewees recounted the degree of fear and concern they felt at the start of the pandemic. A key theme to emerge from the qualitative interviews was the temporal changes in their personal perceptions of risk which generally reduced as the pandemic evolved (online Supporting Information, Table S4, quotes 3–6). This perceived risk reduction was primarily linked to the successful development and introduction of COVID‐19 vaccines, improved patient screening for SARS‐CoV‐2 and the emergence of variants perceived to be less virulent. The few clinicians who reported their perception of risk had not changed since the start of the pandemic tended to be at either end of the self‐identified risk spectrum. They reflected on the changes that had occurred over the previous 2 years but felt their personal position had not altered (online Supporting Information, Table S4, quotes 7–9).

### 3.7 Theme 3: impact on training

*“The training opportunities in anaesthetics are different just because the flow is less so we're able to treat fewer patients.… So the opportunities for training are diminished”* (Æ15).

The impact of the COVID‐19 pandemic on training was frequently raised during interviews and in the survey free‐text comments. One trainee commented that many senior anaesthetists had altered their pre‐pandemic practice to avoid performing AGPs (online Supporting Information, Table S4, quote 1). It was recognised at all training levels that training had been impacted by RPE use as there was decreased healthcare efficiency and a reduced number of patients cared for (online Supporting Information, Table S4, quotes 10–16).

### 3.8 Theme 4: professional endorsement of guidelines

*“[Endorsement should] come from a central anaesthetic body, so if they came from the College, or the AAGBI, I think that would be a kind of reasonable group to be saying, “This is what we're believing”’* (Æ6).

Many interviewees felt the Royal College of Anaesthetists and the Association of Anaesthetists had not directly addressed the issues of AGPs despite IPC guidelines having a huge impact on routine anaesthetic practice. Interviewees reported a high level of trust in their professional bodies (especially compared with governmental organisations) and desired their contribution to and/or endorsement of any guideline changes that would impact practice (online Supporting Information, Table S4, quotes 17–19). Overall, most supported the principle that individual practitioners should have a degree of autonomy regarding RPE, at least during a transition period of guideline change and many actively advocated for this (online Supporting Information, Table S4, quotes 20–24). Some supported a national guidelines framework that could be adapted at hospital level during implementation, but a minority felt strongly that national guidance should preclude hospital or individual variation, as that would lend itself to confusion and potential conflict (online Supporting, Information Table S4, quotes 25–28).

## 4 Discussion

This study has demonstrated good support for the UKHSA IPC guidelines at national, hospital and personal levels during routine anaesthetic practice. Compliance with these guidelines has required dramatic changes to practice. Most respondents felt the IPC measures protected against occupational SARS‐CoV‐2 transmission and allowed patients to be treated without excessive fear for their own health. This survey has uncovered a broad range of perceptions regarding the guidelines and their implementation with substantial variations in practice, including significant deviations from the guidelines. This likely reflects the enormous challenge of implementing the AGP paradigm across the NHS during the pandemic. At the time of the survey, UK IPC policy recommended droplet PPE for all ‘non‐AGP’ patient interactions, irrespective of COVID‐19 status. Our findings indicate that many anaesthetists and their hospitals appear to disagree with this guidance, choosing and/or advocating RPE during pre‐operative assessments of patients with COVID‐19.

Many hospitals and anaesthetists have placed the emphasis of risk for SARS‐CoV‐2 airborne transmission on procedures, distorting risk assessments and management plans required for patient care interactions. The UK IPC guidance states “*Airborne precautions are not required for AGPs on patients/individuals if screening, triaging and testing have confirmed the absence of respiratory infection”* [5]. Yet, 40 (12%) of respondents and 63 (20%) of hospitals advocated the use of RPE when intubating the trachea of any patient, even those who are confirmed not to have COVID‐19. Similar proportions of anaesthetists (70%) would wear RPE during tracheal intubation of a patient of uncertain SARS‐CoV‐2 status as for during a pre‐operative assessment of a patient with confirmed SARS‐CoV‐2 infection. These responses imply that many anaesthetists and their hospitals feel the process of tracheal intubation greatly amplifies the risk of airborne SARS‐CoV‐2 transmission in a patient of uncertain SARS‐CoV‐2 status or even paradoxically in a patient without SARS‐CoV‐2.

Over half of anaesthetists and their hospitals advocated RPE during supraglottic airway device use. Most felt insertion of a supraglottic airway device was a higher risk for SARS‐CoV‐2 transmission than performing a pre‐operative assessment in the same patient. These findings also support the previous report demonstrating 40% of UK hospitals are managing supraglottic airway device use as an AGP [21]. Although their use is not defined as an AGP by either UKHSA or the World Health Organization, it was recommended to be managed as an AGP in guidelines produced by expert consensus in the UK [22]. Additionally, we found compliance with other components of RPE (such as visors) has diminished over the duration of the pandemic. These examples indicate that clinicians and their organisations are implementing IPC guidance by considering their own interpretation of the evidence and personal beliefs.

There is strong evidence that the implementation of UK IPC guidance has posed a challenge to the safe delivery of efficient healthcare resulting in decreased operating theatre utilisation and numbers of patients treated, impaired communication and teamworking, and increased stress and the risk of errors. There are concerns that the increased use of RPE is causing unacceptable and unwarranted environmental harm, as much of the equipment is single use and non‐recyclable. Another theme that emerged from the qualitative study is the impact on teaching and training of anaesthetists in terms of both reduced numbers of cases experienced and the issues of adequate communication while wearing RPE. There has yet to be a cost–benefit healthcare‐economic evaluation of the massive investment in RPE. The factors identified in this study should serve as a counterbalance to any assessment of the benefit from these infection control interventions.

There is recognition that risk perception and personal beliefs have evolved since the first COVID‐19 wave, as understanding of the disease has evolved and new evidence has emerged. While most perceived that the risk associated with AGPs had decreased, the perceptions of some were fixed. This was particularly evident for anaesthetists who identified themselves at either end of the personal risk spectrum. Many expressed frustration that the AGP list and the associated IPC guidelines were not being updated as evidence accumulated during the pandemic. This was especially clear for the degree of aerosol generation from many procedures, the apparent lack of recognition of aerosol generation with coughing, the changed understanding of the different modes of transmission of SARS‐CoV‐2 and the impact of healthcare worker vaccination on the balance of risks.

A substantial number of anaesthetists were keen to not wear RPE during tracheal intubation; however, there were at least as many who expressed a degree of anxiety regarding AGP precaution de‐escalation. Most felt there should be an allowance made for individual practice if anaesthetic procedures are to be removed from the UK AGP list. There was a strong desire for the professional bodies to have input into, or ratify, UK AGP guidance for anaesthetic procedures, as respondents felt these bodies carry considerable weight in shaping the opinions of anaesthetists and other healthcare professionals.

The study has several limitations. The data represent the opinions of anaesthetists between November 2021 and February 2022, and we are aware there has been an evolution of opinions, beliefs and practices throughout the pandemic. The survey was conducted as the Omicron variant was beginning to circulate the UK, before its increased transmissibility, vaccine evasion and somewhat reduced virulence were understood. This was following a period of heightened concern regarding the Delta variant. The qualitative interviews were conducted during the period when Omicron BA.1 was the most common variant. All these factors may have had an impact on perception of COVID‐19 severity. We had a low survey response rate and this may reflect an element of survey fatigue and survey distribution during a late phase of the pandemic, yet respondent characteristics are representative of the UK anaesthetic workforce and the subsequent qualitative interviews have uncovered a wealth of information and insights that could not be determined by the survey alone. It is conceivable that anaesthetists with more polarised views were motivated to complete the survey and respond to the qualitative study invite. Nevertheless, it was those views that we sought to gauge the spectrum of opinions within the profession.

Perceptions regarding AGPs and personal risk from COVID‐19 are evolving rapidly, as is the evidence around aerosol generation and the risks of transmission. The first major evidence‐based revision of a national AGP list for England [23] has recently been published and this new guidance came into effect on 28 May 2022. This removes most of the common airway management procedures from the list including tracheal intubation, tracheal extubation and facemask ventilation. It also provides clarification that supraglottic airway device use is not considered an AGP. In this new phase of evidence‐based IPC guideline updates, it is important that the prevailing attitudes to AGPs, RPE, modes of transmission and personal risk of anaesthetists are factored into the plans for dissemination and implementation of the changes.

We are not aware of any previous survey of anaesthetists' attitudes to AGPs; therefore, this study has provided important behavioural insights into the barriers, enablers and drivers that could facilitate successful communication and implementation of the new guidance. Any future guidance changes should have input from groups representing the clinical specialties affected, and ideally have endorsement by professional bodies. It is important that the group of anaesthetists that still identify as high risk, and those who are anxious about changes to practice, are supported through any phase of de‐escalation of airborne precautions, especially while the prevalence of COVID‐19 remains high in the community.

## Supporting information


**Appendix S1.** Original survey questions and link.

**Table S1.** Awareness and perceptions of aerosol‐generating procedure guidelines.
**Table S2.** Impact of COVID aerosol‐generating procedure guidelines on practice.
**Table S3.** Personal perception of risk.
**Table S4.** Emergent qualitative themes.
