# Supplementary material for: Anaesthetists' current practice and perceptions of aerosol‐generating procedures: a mixed‐methods study
Source: Anaesthesia. 2022 Jul 21;77(9):959–70. doi: 10.1111/anae.15803 (PMC9543704; doi:10.1111/anae.15803)
Supplement: Supplementary file 1 — Appendix S1. Original survey questions and link. [file ANAE-77-959-s002.pdf]

Survey used to determine: Anaesthetists' perceptions of Aerosol Generating Procedures

This survey aims to examine current practice and anaesthetists' perceptions regarding airway management in the context of COVID-19.

We are looking specifically at high risk **Aerosol Generating Procedures**, defined by Public Health England and the WHO as:

" ... medical procedures that have been reported to be aerosol-generating and consistently associated with an increased risk of pathogen transmission"

The WHO currently define aerosol as droplets <5μm in diameter. These aerosols are respirable and able to deposit deep within the human respiratory tract.

It should take less than **10 minutes** to complete this survey

This survey is part of the NIHR funded **MAGPIE** study (NIHR301520) undertaken in conjunction with the NIHR funded **AERATOR** study (COV0333). **AERATOR** has been conducting research investigating aerosol generation from medical procedures.
If you require any further information, please contact andy.shrimpton@bristol.ac.uk

This survey is for Anaesthetists who are currently practising / performing routine anaesthetic care

Please note this survey is no longer live and any responses will not be counted. "Skip logic" has been removed from some questions to enable all questions to be viewed

Introduction

This survey is **anonymous** and aims to determine **your opinions** regarding the risks of COVID-19 and AGPs.
Your participation is greatly appreciated.

I understand that my participation is completely voluntary *

- No
- Yes

I would like to take part in this study *

- No
- Yes

Hospital Policy

Does your hospital have a policy for COVID-19 infection control precautions during anaesthetic airway management / AGPs? *

- Yes
- No
- Unsure

Scenario 1

You are required to undertake a pre-operative assessment of a symptomatic COVID-19 POSITIVE patient in a side room

PPE = personal protective equipment
Droplet PPE = fluid resistant surgical mask (non respirator), apron, gloves and eye protection
Airborne PPE = droplet PPE plus a fitted respirator type mask (FFP3/Hood) and fluid repellent gown

What COVID-19 precautions does your HOSPITAL mandate for ASSESSMENT of this patient? *

- Airborne PPE
- Droplet PPE
- Unsure / Don't know

What COVID-19 precautions do YOU feel are appropriate for ASSESSMENT of this patient? *

- ◯ Airborne PPE
- ◯ Droplet PPE
- ◯ Unsure / Don't know

Which mode(s) of COVID-19 transmission are you MOST concerned about in this scenario? *

Select each that you think apply

- ☐ Airborne particles (<5μm diameter)
- ☐ Droplet (drops >5μm diameter)
- ☐ Fomite (virus on solid objects)
- ☐ Direct contact with secretions/fluids
- ☐ Unsure / Don't know

Scenario 2

You assess a patient admitted on the ward who has been admitted for urgent surgery.

The patient is asymptomatic for COVID-19, has not self-isolated and has a PCR test result pending

What COVID-19 precautions does your HOSPITAL mandate for pre-operative ASSESSMENT of this patient? *

- ◯ Airborne PPE
- ◯ Droplet PPE
- ◯ Unsure / Don't know

What COVID-19 precautions do YOU feel are appropriate for pre-operative ASSESSMENT of this patient? *

- ◯ Airborne PPE
- ◯ Droplet PPE
- ◯ Unsure / Don't know

The same patient needs an emergency appendicectomy.

The patient is asymptomatic for COVID-19, has not self-isolated and has a PCR test result pending

---

What COVID-19 precautions does your HOSPITAL mandate for INTUBATION of this patient? *

- ◯ Airborne PPE
- ◯ Droplet PPE
- ◯ Unsure / Don't know

Is fallow time mandated after intubation? *

Fallow time = time to allow aerosol to settle before movement can occur in / out of the room

- ◯ Yes
- ◯ No
- ◯ Unsure / Don't know

What COVID-19 precautions do YOU feel are appropriate for INTUBATION of this patient? *

- ◯ Airborne PPE
- ◯ Droplet PPE
- ◯ Unsure / Don't know

Do you feel fallow time is needed after intubation? *

Fallow time = time to allow aerosol to settle before movement can occur in / out of the room

- ◯ Yes
- ◯ No
- ◯ Unsure / Don't know

Which mode(s) of COVID-19 transmission are you MOST concerned about in this scenario? *

Select each that you think apply

- ☐ Airborne particles (<5µm diameter)
- ☐ Droplet (drops >5µm diameter)
- ☐ Fomite (virus on solid objects)
- ☐ Direct contact with secretions/fluids
- ☐ Unsure / Don't know
- ☐ I am not concerned

What level of risk do you feel intubating this patient poses for COVID-19 transmission compared to speaking with them pre-operatively? *

- ◯ Much higher
- ◯ Higher
- ◯ An equal risk
- ◯ Lower
- ◯ Much Lower

If the patient's PCR test was negative, they had fully self-isolated for 14 days prior to admission and were asymptomatic for COVID-19 (i.e. a 'Green' patient)

What COVID-19 precautions does your HOSPITAL mandate for INTUBATION of this patient? *

- ◯ Airborne PPE
- ◯ Droplet PPE
- ◯ Unsure / Don't know

What COVID-19 precautions do YOU feel are appropriate for INTUBATION of this patient? *

- ◯ Airborne PPE
- ◯ Droplet PPE
- ◯ Unsure / Don't know

Scenario 3

You decide to use a SUPRAGLOTTIC AIRWAY for a patient requiring incision and drainage of an axillary abscess.

The patient is asymptomatic for COVID-19, has not self-isolated and has a PCR test result pending

What COVID-19 precautions does your HOSPITAL mandate for SUPRAGLOTTIC AIRWAY use in this patient? *

- ◯ Airborne PPE
- ◯ Droplet PPE
- ◯ Unsure / Don't know

Which of the following are also mandated when using a supraglottic airway device in this patient? *

- ☐ Device removal only in theatre
- ☐ Fallow time after device insertion and removal
- ☐ None
- ☐ Unsure / Don't know

What COVID-19 precautions do YOU feel are appropriate for SUPRAGLOTTIC AIRWAY use in this patient? *

- ◯ Airborne PPE
- ◯ Droplet PPE
- ◯ Unsure / Don't know

Which of the following do you feel should also be employed when using a SUPRAGLOTTIC AIRWAY in this patient? *

(select all that apply)

- ☐ Device removal only in theatre
- ☐ Fallow time after device insertion and removal
- ☐ None
- ☐ Unsure / Don't know

Which mode(s) of COVID-19 transmission are you MOST concerned about in this scenario? *

Select each that you think apply

- ☐ Airborne particles (<5μm diameter)
- ☐ Droplet (drops >5μm diameter)
- ☐ Fomite (virus on solid objects)
- ☐ Direct contact with secretions/fluids
- ☐ Unsure / Don't know
- ☐ I am not concerned

What level of risk do you feel SUPRAGLOTTIC AIRWAY use in this patient poses for COVID-19 transmission compared to speaking with them pre-operatively? *

- ○ Much higher
- ○ Higher
- ○ An equal risk
- ○ Lower
- ○ Much Lower

Infection Control Practices

I believe the infection control procedures in my immediate work environment are sufficiently effective to prevent the spread of COVID-19 *

Strongly disagree | Disagree | Somewhat disagree | Neither agree nor disagree | Somewhat agree | Agree | Strongly agree

If you disagree, please state which aspects you feel are INSUFFICIENT to prevent COVID-19 transmission *

(Select all that apply)

- [ ] Testing of staff
- [ ] Testing of patients
- [ ] Fallow time between AGPs
- [ ] Adherence of STAFF to infection control procedures (eg. mask wearing / social distancing)
- [ ] Adherence of PATIENTS / VISITORS to infection control procedures (eg. mask wearing / social distancing)
- [ ] Patient self-isolation prior to admission
- [ ] Cohorting COVID-19 positive patients
- [ ] Availability of airborne PPE
- [ ] Other

Please state *

Following recommended infection, prevention and control procedures adds significant additional strain to my workload *

Strongly disagree | Disagree | Somewhat disagree | Neither agree nor disagree | Somewhat agree | Agree | Strongly agree

I feel the additional strain is appropriately balanced against the risks of COVID-19 transmission *

Strongly disagree | Disagree | Somewhat disagree | Neither agree nor disagree | Somewhat agree | Agree | Strongly agree

Impact of airborne COVID-19 precautions

To what extent do you agree with the following statements about impact?

Airborne infection prevention control measures implemented for COVID-19 have :

DECREASED operating list turnover *

Strongly disagree | Disagree | Somewhat disagree | Neither agree nor disagree | Somewhat agree | Agree | Strongly agree

IMPROVED communication with patients *

Strongly disagree | Disagree | Somewhat disagree | Neither agree nor disagree | Somewhat agree | Agree | Strongly agree

Significantly REDUCED my risk of contracting COVID-19 *

Strongly disagree | Disagree | Somewhat disagree | Neither agree nor disagree | Somewhat agree | Agree | Strongly agree

INCREASED the risk of making clinical errors *

Strongly disagree | Disagree | Somewhat disagree | Neither agree nor disagree | Somewhat agree | Agree | Strongly agree

Made team working MORE DIFFICULT *

Strongly disagree | Disagree | Somewhat disagree | Neither agree nor disagree | Somewhat agree | Agree | Strongly agree

DECREASED stress and anxiety for healthcare workers *

Strongly disagree | Disagree | Somewhat disagree | Neither agree nor disagree | Somewhat agree | Agree | Strongly agree

Resulted in an UNACCEPTABLE environmental cost *

Strongly disagree | Disagree | Somewhat disagree | Neither agree nor disagree | Somewhat agree | Agree | Strongly agree

INAPPROPRIATELY allocated healthcare resources *

Strongly disagree | Disagree | Somewhat disagree | Neither agree nor disagree | Somewhat agree | Agree | Strongly agree

Perceptions

Please specify if you agree or disagree with the following

I am concerned about the risk to myself of becoming ill with COVID-19 *

Strongly disagree | Disagree | Somewhat disagree | Neither agree nor disagree | Somewhat agree | Agree | Strongly agree

I am concerned about the risk to my family related to COVID-19 as a result of my job role *

Strongly disagree | Disagree | Somewhat disagree | Neither agree nor disagree | Somewhat agree | Agree | Strongly agree

I am afraid of looking after patients who are ill with COVID-19. *

Strongly disagree | Disagree | Somewhat disagree | Neither agree nor disagree | Somewhat agree | Agree | Strongly agree

Personal Risk

Have you had COVID-19? *

- Yes
- No
- Prefer not to say
- Unsure / Don't know

Have you had a COVID-19 vaccination? *

- Yes (initial course complete)
- Yes (initial course not complete)
- No
- Prefer not to say

Have you had a booster jab?

- Yes
- No
- Prefer not to say

Prior to this Vaccination, based on your demographics (age, gender, BMI, ethnicity, pre-existing health conditions) what risk did you consider COVID-19 posed to your health? (0 = low risk, 100 = highest risk) *

0 100

Based on your demographics (age, gender, BMI, ethnicity, pre-existing health conditions), what risk did you consider COVID-19 posed to your health? (0 = low risk, 100 = highest risk) *

0 100

Are you in either the 'at risk' or 'clinically extremely vulnerable' groups? *

- ◯ Yes
- ◯ No
- ◯ Prefer not to say
- ◯ Unsure / Don't know

Risks to Healthcare Workers

What level of risk should HEALTHCARE WORKERS be exposed to, from COVID-19, during routine clinical practice? *

Compare to the risk faced by other keyworkers eg. police officers, firefighters, transport workers, teachers

◯ Much lower ◯ Slightly lower ◯ Equivalent ◯ Slightly higher ◯ Much higher

What level of risk do you think ANAESTHETISTS face of contracting COVID-19, compared to other healthcare workers, during routine clinical practice? *

◯ Much lower ◯ Slightly lower ◯ Equivalent ◯ Slightly higher ◯ Much higher

Guidance

If there was a change of national guidance, how would you feel about not wearing airborne PPE during intubation of an asymptomatic patient of uncertain COVID status? *

- ◯ Extremely anxious
- ◯ Mildly anxious
- ◯ Ambivalent
- ◯ Comfortable
- ◯ Keen to move away from airborne PPE

What are you most concerned about? *

- ☐ Lack of evidence demonstrating safety
- ☐ Lack of trust in Public Health England / WHO guidelines
- ☐ Other

Please state *

We are very keen to understand and explore perceptions, anxieties and concerns related to de-escalating PPE use. Would you be happy to be contacted to further explore your perceptions and feelings? *

- ◯ Yes
- ◯ No

Please provide your email address if happy to be contacted *

Experience

We are interested in defining the impacts that airborne PPE and the associated infection control procedures have had on your ability to deliver anaesthetic care during the COVID-19 pandemic.

If you would like to share your experience (either positive or negative) please state below

We are interested whether your airway management practice has changed during the COVID-19 pandemic

If you would like to share how your airway management practice has changed, please state below

(Please do NOT include patient identifiable information)

Demographics

This survey is anonymous, we want to collect this information to ensure a representative sample of the anaesthetic workforce is obtained

In which country of the UK do you work? *

- ◯ England
- ◯ Scotland
- ◯ Wales
- ◯ Northern Ireland

Which area of the England are you based? *

- North East
- North West
- Yorkshire and the Humber
- East Midlands
- West Midlands
- East of England
- London
- South East
- South West

Which best describes the facility you work in the majority of the time? *

- Tertiary Referral Centre
- University Teaching Hospital
- District General Hospital
- Day case procedure centre
- Private hospital / surgical unit
- Other

Please state

What is your primary position / role? *

- ◯ Consultant level doctor (or post CCT fellow)
- ◯ Associate Specialist / SAS
- ◯ Registrar (ST3-ST8)
- ◯ Core Trainee (CT1-CT3)
- ◯ ACCS (anaesthesia only)
- ◯ ACCS (non-anaesthesia)
- ◯ Clinical / Research Fellow
- ◯ LATs (Locum Appointment for Training)
- ◯ LAS (Locum Appointment for Service)
- ◯ MTI (Medical Training Initiative)
- ◯ Trust Doctor
- ◯ Other

Please state

....

What best describes your gender? *

- ◯ Female
- ◯ Male
- ◯ Non-Binary
- ◯ Prefer Not To Say
- ◯ Prefer to self Identify

Please state *

....

What is your age? *

- ◯ 20-29
- ◯ 30-39
- ◯ 40-49
- ◯ 50-59
- ◯ 60-69
- ◯ 70-79
- ◯ 80+
- ◯ Prefer not to say

What is your ethnicity? *

- ◯ White
- ◯ Mixed/Multiple ethnic groups
- ◯ Asian /Asian British
- ◯ Black / African / Caribbean / Black British
- ◯ Other ethnic group
- ◯ Prefer not to say

Any other comments or thoughts?

Thank you very much for you time
