# Supplementary material for: Anaesthetists' current practice and perceptions of aerosol‐generating procedures: a mixed‐methods study
Source: Anaesthesia. 2022 Jul 21;77(9):959–70. doi: 10.1111/anae.15803 (PMC9543704; doi:10.1111/anae.15803)
Supplement: Supplementary file 2 — Table S1. Awareness and perceptions of aerosol‐generating procedure guidelines. Table S2. Impact of COVID aerosol‐generating procedure guidelines on practice. Table S3. Personal perception of risk. Table S4. Emergent qualitative themes. [file ANAE-77-959-s001.docx]

**Table S1** Awareness and perceptions of aerosol-generating procedure guidelines. Quotes from anaesthetists during interviews (Æ1-18) or extracted from “free text” (FT) sections of the survey.

| Awareness and perceptions of aerosol-generating procedure guidelines | | |
| --- | --- | --- |
| Quote# | Interviewee | Quote |
| 1 | Æ5 | “Am I rule follower or a rule breaker? I’m a rule follower, I think most of us aren’t we at the bottom line.” |
| 2 | Æ5 | “FFP3 type protection although was deeply unpleasant for long periods of time, I think that did protect us.” |
| 3 | Æ8 | “I've got ideas of what I think I should do but I think the same point you're in a leadership position, a clinical leadership position so you need to represent the policy.” |
| 4 | Æ10 | “Different people in the same room [are] wearing different levels of PPE… but you can see how in an evolving situation over a long period of time… that happens... People that were fed up with wearing PPE just weren’t wearing full PPE. People like me that felt, well I’d better do it because the theatre staff are telling me I should, and I don’t want conflict, and I think I should be seeing to be doing what they expect.” |
| 5 | Æ15 | “I think [AGP guidelines are] very out of date for what the evidence is now around what is an AGP and what isn’t an AGP. And also taking into account the fact that we’re swabbing people and vaccination status” |
| 6 | FT21 | “Intubation is not an AGP. Coughing produces more aerosols. Poor staff on wards have to wear surgical masks while walking around in a room full of aerosols. How the guidance has not been changed nationally, I do not know.” |
| 7 | FT83 | “The mandated rules in theatre are not adhered to. I feel this is because IPC have completely lost the faith of employees. Rules have changed so frequently, and one hospital has different rules to another a few miles away with identical population Covid numbers.” |
| 8 | Æ2 | “I think NHS England should have made it clear what we should or shouldn’t be doing and it will leave less of it open to interpretation, because each unit has been interpreting it differently” |
| 9 | Æ8 | “I think that's why we've got confusion at the moment is because lots of people are doing their own thing. No one really knows the evidence for these things individually |
| 10 | Æ14 | “I do think intubation is very high risk for aerosol generation, nearly going right into the mouth of the patient” |
| 11 | Æ8 | “I've always got a low threshold to put on FFP3, so even if they're not COVID and in respiratory failure and I don't know why, I put on FFP3…” |
| 12 | FT73 | “I do wear PPE even now, and for green patients. I have had Covid once, and under no circumstances would wish to repeat that” |
| 13 | Æ16 | “I mean yes straightforward intubation possibly I can understand why you would say it may not be an aerosol generating procedure. But if it becomes a difficult intubation and it has to be done twice, twice, bad vents in the middle of it and so then it’s, you kind of increase the risk, so yes [it is an AGP]” |
| 14 | Æ10 | ‘I think now the only reason I think we maybe at slightly higher risk is because we do get very close to people because you have to be close to someone to anaesthetise them’ |
| 15 | Æ13 | “I think some of our advanced airway procedures are potentially risky but not for the reasons that we first thought. So, for example intubation per se, inserting and removing supraglottic airways per se, I don’t think that that’s high risk at all” |
| 16 | Æ2 | ‘Well the point that we’re intubating someone, they’re not breathing, it’s sort of a negative AGP’. |
| 17 | FT283 | “Aerosol PPE should be policy for symptomatic COVID patients, irrespective of intubation procedure. A cough generates more aerosol than a tube.” |
| 18 | Æ1 | “I think if I am speaking face to face with somebody, I think there is as much aerosol being generated as inserting an LMA or maybe even a tube.” |
| 19 | Æ13 | “But certainly not intubation and inserting supraglottic airways especially if you don’t bag-mask the patient… even now I’m going with the flow and we’re wearing FFP3 masks etc, I don’t, I honestly don’t see those procedures as major high-risk compared to just talking to the patient close range.” |
| 20 | Æ6 | “There’s very, very few patients that were coming through without some form of screening, so we had a pretty good idea about whether or not you had it. And if you don’t have COVID you’re no risk to me at all. If you do have COVID most of the time we had a good idea” |

**Table S2** Impact of COVID aerosol-generating procedure guidelines on practice. Quotes from anaesthetists during interviews (Æ1-18) or extracted from “free text” (FT) sections of the survey

| Quote# | Interviewee | Quote |
| --- | --- | --- |
| 1 | Æ14 | “It was quite uncomfortable then with the loud sound, you are shouting at each other, and you know ..so… it was like quite distressing at times” |
| 2 | Æ5 | “A big part of what we do every day is working as a team and you can’t be a team player if you can’t talk to people” |
| 3 | Æ9 | “I feel there is a major adverse event just waiting to happen… going to say something that doesn’t get heard and someone’s going to suffer because communication is significantly impaired” |
| 4 | Æ5 | “You ask your assistant to go and get something and they don’t understand properly what you said, it’s dangerous.” |
| 5 | Æ10 | “I deal with the critical incident submission forms that come from my department, and there were definitely a couple of crucial incidents where people cited communication through PPE as a factor in management of the situations.” |
| 6 | Æ5 | “Our productivity has probably halved, probably worse than that. It’s made massive changes to … our day-to-day working lives”. |
| 7 | Æ9 | “I think there’s probably not enough attention given to how AGPs impact our productivity and therefore it has an impact on waiting lists and all that. I think that’s all been brushed under the carpet” |
| 8 | Æ9 | “It has got some positives. Now, if we need to intubate and we need to wait the ten minutes, it’s actually quite a nice ten minutes where no-one’s bothering you. The surgeon’s not on your back, you’ve got time to make sure you’re properly set up, you’re good to go, you’ve given all your drugs, put in all the other lines you need. It’s quite a nice ten minutes to just kind of focus. I wouldn’t be opposed if we kept a little dedicated anaesthetic time.” |
| 9 | Æ8 | “There was just massive amount of plastic waste and I don't think much of it is evidence based, so I can understand FFP3 masks but I don't understand why we were all wearing surgical gowns for every patient and we were all wearing double gloves… and then visors on top” |
| 10 | FT186 | “e.g. fogging up of visor causing reduced visibility and potentially increasing intubation time/decreasing the chance of first pass success” |
| 11 | Æ11 | “I used to think probably six months ago thought this is really awful, we can’t keep doing this forever. And now I think I could have an FFP3 mask on for my intubations forever if I had to. Like I feel like I’ve got used to that now.” |
| 12 | Æ13 | “I’m quite happy to wear FFP3 masks for quite prolonged periods actually.” |
| 13 | AE7 | “I think ITU got protected really well and actually we probably didn’t need it. I think the wards were left with inadequate protection. So, I mean with the benefit of hindsight I think we’ve probably had it the wrong way around.” |
| 14 | Æ12 | “I intubated five or six really sick patients with COVID and still didn’t manage to get it. And after that I thought, well actually I do trust the PPE. And I trust the droplet PPE that we wear at work.” |
| 15 | Æ11 | “We had a separate donning room. We walk into there and we don and then go into ITU. And you’re kind of, you feel quite protected. It felt really safe actually.” |
| 16 | Æ11 | “I think compared to the hospital population actually anaesthetists probably are relatively protected.” |
| 17 | Æ11 | “But I more feel for the people... who perceive themselves to be high risk making decisions and are they going to end up thinking, well perhaps anaesthetics isn’t for me because I’m high risk. Or can we make them feel safe by there being clear guidance that these are the risky things, and this is what we do to mitigate that risk? And then they think well I can go to work because my colleagues around me will support me in being safe...” |

**Table S3** Personal perception of risk. Quotes from anaesthetists during interviews (Æ1-18) or extracted from “free text” (FT) sections of the survey

| Quote# | Interviewee | Quote |
| --- | --- | --- |
| 1 | FT152 | “I was concerned about my safety and anxious about carrying the disease home, but at the end of the day, I felt that as doctors we are trained to treat patients whatever may be their disease, so we just have to take the necessary precautions” |
| 2 | Æ18 | “I follow generally what the hospital wants except that if I particularly feel that I’m wearing full PPE, that’s what I’m doing. And if anyone wants to argue with me, they can do it.” |
| 3 | Æ3 | “I have always taken myself to be at high risk you know just because of my ethnicity, from what I was seeing and from what I reading… so I do look at myself as somebody who is vulnerable, so I sometimes take extra precautions.” |
| 4 | Æ15 | “I know some colleagues are on immunosuppressants and things like that. So they are at higher risk. And some colleagues have morbid obesity and things like that so they, I have noticed that they are more likely to be the ones that are wearing the FFP3 even for just sitting in the computer room or something like that.” |
| 5 | Æ11 | So I feel like people’s perception of how high risk it is and how awful it is depends a bit on how far out of their normal like, the sort of work for me felt like it was my normal work. And I guess I’m fortunate that I haven’t had any family members or close friends who have been unwell with COVID at all. So that’s probably helped. |
| 6 | Æ16 | “Yes I’ve had friends who were quite ill. Fortunately I don’t have, I mean my immediate work and friends I haven’t had anyone who’s died from it. But yes, the emotional burden of tending to patients who ordinarily are very pleasant people who just were unfortunate to get this infection and then watching them die has been, sometimes it takes its toll when you think about it” |
| 7 | Æ10 | “And then I found actually after I’d had [COVID] I felt a lot better.” |
| 8 | Æ18 | “They referred me to a long Covid clinic and it’s only when you start doing echoes that you realise that you have a small pericardial effusion... so unless you actually start looking for things, then potentially, you don’t know what your long-term consequence is, if you were exposed to chronic infection” |
| 9 | Æ13 | “I mean I think now people are getting it, it’s extremely common, nearly everybody’s getting it with very mild symptoms particularly if they’ve been vaccinated and boosted. So I would regard it as a pain. And really no more than that...” |
| 10 | Æ16 | “You don’t want to be the one who’s having serious infection and having to be on ICU and needing all that care” |
| 11 | Æ4 | “Over time, you got less worried about death and more about long COVID” |
| 12 | Æ14 | I thought I had a very high risk because first of all I’m a single parent ... I have no like immediate family to help me out you know |
| 13 | Æ1 | “If I get severe COVID and I cannot work? Then I would have to go back home to my country… I am a permanent resident here, but I would have to go back because I couldn’t stay.” |
| 14 | Æ10 | “I would actually see the children as a bigger risk factor than work [for transmitting COVID-19]” |
| 15 | FT83 | “It changed at the start, but it’s slackened considerably (to a FRSM) as I witnessed that healthcare infections didn’t really seem to be caused by patient to healthcare worker transmission; the overwhelming majority came from family and community transmission just like our patients” |

**Table S4** Emergent qualitative themes. Quotes from anaesthetists during interviews (Æ1-18) or extracted from “free text” (FT) sections of the survey

| Quote# | Interviewee | Quote |
| --- | --- | --- |
| **Change in anaesthetic practice** | | |
| 1 | Æ9 | “A lot of people [adapted] their practices to try to circumvent AGPs… it’s been very, very confusing for me.” |
| 2 | FT281 | “I now actively look for opportunities to use regional/ non GA based techniques to avoid the need for AGPs” |
| **Change in perceptions over time** | | |
| 3 | Æ5 | “The Italian intensivists were seen to be falling apart on live TV almost and that was shocking and greatly concerning and we also thought this terrible thing was coming to us and no one really knew what your personal risk was going to be” |
| 4 | Æ5 | “The Italian intensivists were seen to be falling apart on live TV almost and that was shocking and greatly concerning and we also thought this terrible thing was coming to us and no one really knew what your personal risk was going to be” |
| 5 | Æ10 | “I think initially, when we didn’t know so much about COVID, I think it was felt that we were at much higher risk. And now that we have more information I’m not sure that’s necessarily true.” |
| 6 | Æ4 | ‘What I’ve learned over time is that I’m probably a bit more relaxed about it as well. I think at the start, I think everybody was a bit like, ‘I don’t want to stick a tube in anybody, I’m going to die from Covid,’ which is probably extreme” |
| 7 | Æ9 | “I remember at the beginning of the pandemic, this was sort of May [2020]… people coming out with case fatalities and ratios, statistics on how actually dangerous COVID is, based on age and all that… I figured at that point, I was more at risk in my daily commute to a DGH, than I was from COVID.” (perceived personal risk 0/100) |
| 8 | Æ17 | “I’m in the demographic to (a) catch it; and (b) die from it… I still take it very seriously…” “[My risk perception] has probably stayed the same. I mean what’s changed is vaccinations and treatments and possibly Omicron is not as bad. If you’re vaccinated, very likely it’s not as bad.” (perceived personal risk 76/100) |
| 9 | Æ15 | “But definitely my perception is that it’s…. We’re in a less risky state now mostly due to vaccination, partly due to the change in the variants.” |
| **Impact on training** | | |
| 10 | Æ15 | “The training opportunities in anaesthetics are different just because the flow is less so we’re able to treat fewer patients.… So the opportunities for training are diminished.” |
| 11 | Æ7 | “…the number of cases you get through has gone” |
| 12 | Æ16 | “Well, it’s meant some change to rotations now and then and what the expected outcomes from each rotation had to be adjusted a bit... they’ve had to change how many patients you needed to have seen to get signed off because obviously there are lots of disruptions to the service… And so clinical experience may have been somewhat affected adversely” |
| 13 | Æ5 | “… there’s a whole cohort of trainees now because they rotate through quite frequently, I only know what they look like from here to here because I’ve never seen the rest of them and it’s very hard when you’re only seeing a small proportion of someone’s face, it does impede communication and trust. When you’re sat trying to have a teaching session with someone, for example, it is more difficult, you lose a lot of non-verbal clues when all you can see is some eyes” |
| 14 | Æ12 | “I remember trying desperately to do a case-based discussion at 4 in the morning during an laparotomy on a Covid positive patient, when we just used to wear the PPE for the whole case, because none of us knew quite what was aerosol generating and quite what wasn’t. And it was awful. And you couldn’t teach” |
| 15 | FT68 | “Significantly prolonged the time taken to complete each case, therefore reduced number of operations and therefore reduced exposure to training lists and numbers for my training” |
| 16 | FT137 | “I started my core training in the beginning of COVID and I had little practice on putting tea-bag on LMA and seeing patients wake up naturally in recovery because we took always the LMA out in the theatre” |
| **Professional endorsement of guidelines** | | |
| 17 | Æ6 | “[Endorsement should] come from a central anaesthetic body, so if they came from the College, or the AAGBI, I think that would be a kind of reasonable group to be saying, “This is what we’re believing.” |
| 18 | Æ1 | “You can’t have somebody else deciding for the anaesthetists what seems to be a risk, you know, what seems to be a risky thing for them.” |
| 19 | Æ15 | “People are often reluctant to change… they might perceive that we’re changing because we want to improve flow and for non-clinical reasons rather than evidence-based clinical reasons.” |
| 20 | Æ6 | “I suspect what we should have is an awful lot more flexibility, that we say you know, “If you’re comfortable wear it, if you’re not comfortable and you want to wear proper PPE it is available, it’s here”, you know there is no pressure to not wear it. Because I think a lot of the problem is anxiety, you know our recovery still will not take patients with a Guedel in, or a tube, or an LMA, because they’ve decided that they’re all extremely vulnerable to getting COVID.” |
| 21 | Æ2 | “If one team member says I’m anxious about this process, you have to accept that their anxiety means, you have to manage, you have to do whatever it is that they think is safe for them as well” |
| 22 | Æ17 | “I would feel mildly unhappy [if intubation was removed from the AGP list]… I would see the reasoning behind it… I would probably do what I wanted to do anyway.” |
| 23 | Æ14 | “Everyone needs to take care of their health and some responsibility and be able to have that freedom to feel that what they feel is comfortable and right for them.” |
| 24 | Æ7 | “The big barrier to [change] is going to be people. Their perception of the risk. And their perception of what their personal risk is as well’ |
| 25 | Æ10 | “I think it’s useful to have national standards that local guidelines can then refer to… sometimes what works in one locality doesn’t work in another.” |
| 26 | Æ10 | “Guidelines… work best if there’s a sort of national or international standard that is then locally implemented in a way that is relevant to the locality.” |
| 27 | Æ8 | “[I’m] going to sound a little authoritarian, but I don't think there should be [individual variation]… it just creates a mess. I think that's why we've got confusion at the moment is because lots of people are doing their own thing” |
| 28 | Æ16 | “It’s hard to say go with individual circumstances because then you would always have the cowboys and misanthrope who would like say, I don’t need anything... So there would be a bit of peer pressure then in the middle of it… it’s probably best to have same standards rather than varying it per individual” |
